# Krüppel‐Like Factor 4, a Hub Gate for Cell Crosstalk in Tumor Microenvironment

**DOI:** 10.1002/cam4.71498

**Published:** 2026-01-14

**Authors:** Min Tang, Binle Tian, Jingyi Zhou, Di Ma, Rongze Sun, Qi Li

**Affiliations:** ^1^ Department of Medical Oncology, Cancer Center Shanghai General Hospital, Shanghai Jiao Tong University School of Medicine Shanghai China

**Keywords:** cancer, immune cells, KLF4, tumor microenvironment

## Abstract

**Objective:**

Krüppel‐like factor 4 (KLF4) is a zinc finger transcription factor that plays context‐dependent roles in cancer. It functions as either a tumor suppressor or an oncogene depending on tumor type and cellular context. This review aimed to comprehensively summarize the roles of KLF4 in the tumor microenvironment (TME) and evaluate its potential as a therapeutic target.

**Methods:**

We conducted a comprehensive literature review to elucidate the expression patterns, regulatory mechanisms, and functional roles of KLF4 across different TME components, including cancer cells, immune cells, cancer‐associated fibroblasts, pericytes, and extracellular matrix.

**Results:**

KLF4 exhibits dual roles in cancer cells, acting as a tumor suppressor in gastric, lung, and pancreatic cancers while promoting oncogenesis in breast, colorectal, and prostate cancers. In the TME, KLF4 regulates macrophage polarization (M1/M2), T‐cell exhaustion, NK cell activity, and MDSC recruitment. Additionally, KLF4 modulates CAF activation and ECM remodeling. KLF4 expression is regulated by miRNAs, lncRNAs, and epigenetic modifications. Emerging therapeutic strategies targeting KLF4, such as APTO‐253, show promise in preclinical and early clinical trials.

**Conclusions:**

KLF4 serves as a hub gate orchestrating cell crosstalk within the TME. Understanding its context‐dependent functions may facilitate the development of KLF4‐targeted therapies for precision oncology.

## Graphical abstract

Krüppel‐like factor 4 (KLF4) exhibits a dual, context‐dependent role, acting as both a tumor suppressor and an oncogene. It remodels the tumor microenvironment by modulating cancer, stromal, and immune cells, representing a promising yet complex therapeutic target for cancer treatment.

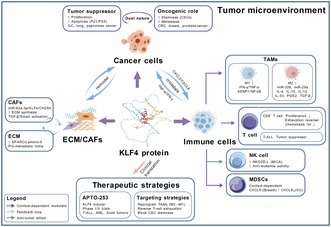

**Abbreviations**
5Z7O: 5Z‐7‐oxozeaenol
AHR: aryl hydrocarbon receptor
ATF2: activating transcription factor 2
CAFs: cancer‐associated fibroblasts
CagA: cag pathogenicity island protein A
CH25H: cholesterol 25‐hydroxylase
CHL: classical Hodgkin's lymphoma
circRNA: circular RNA
CLL: chronic lymphocytic leukemia
CRC: colorectal cancer
CSC: cancer stem cell
CTL: cytotoxic T lymphocyte
DCs: dendritic cells
DLT: dose‐limiting toxicities
ECM: extracellular matrix
FSP‐1: fibroblast‐specific protein‐1
GC: gastric cancer
HCC: hepatocellular carcinoma
iPSC: induced pluripotent stem cells
JNK: c‐Jun NH(2)‐terminal kinase
KLF4: Krüppel‐like factor 4
lncRNAs: long noncoding RNAs
MDS: myelodysplasia
MDSCs: myeloid‐derived suppressor cells
miRNAs: microRNAs
MPN: myeloproliferative neoplasms
MTD: maximum tolerated dose
MUC5AC: mucin 5 AC
NHL: non‐Hodgkin's lymphoma
NLSs: nuclear localization signals
SAE: serious adverse events
SENP1: specific peptidase 1
SUMO: small ubiquitin‐like modifier
T‐ALL: T‐cell acute lymphoblastic leukemia
TAMs: tumor‐associated macrophages
Th17: T helper cell 17
TME: tumor microenvironment
TNBC: triple‐negative breast cancer
UTR: untranslated region
YY1: Yin‐Yang 1

## 1 Introduction

Krüppel‐like factor 4 (KLF4) is one of the most reported members of the evolutionarily conserved family of zinc finger transcription factors, first identified in 1996 [1]. Initially, KLF4 was characterized as a pivotal regulator of diverse cellular processes, including development, differentiation, proliferation, somatic cell reprogramming, tissue homeostasis and apoptosis [2]. Recognized primarily for its role as one of the Yamanaka factors, KLF4 is indispensable for maintaining the pluripotency of embryonic stem cells and the induction of induced pluripotent stem cells (iPSC) [3]. Beyond stem cell biology, KLF4 has been implicated in the pathogenesis of inflammation and cancer, exhibiting a context‐dependent and sometimes paradoxical role [4, 5]. For example, the expression of KLF4 is decreased in gastric cancer (GC) and typically exerts a tumor‐suppressive role [6]. However, in breast cancer, KLF4 exhibits both tumor‐suppressive and oncogenic activities [7, 8].

In recent years, increasing attention has shifted toward viewing these seemingly contradictory roles of KLF4 through the lens of the tumor microenvironment (TME). The TME—comprising immune cells, stromal cells, extracellular matrix (ECM), and soluble factors—has emerged as a central regulator of every stage of tumorigenesis, from initiation to metastasis [9]. Rather than acting solely within tumor cells, KLF4 exerts many of its effects indirectly by shaping the behavior of TME components. Indeed, TME‐derived cues influence KLF4 expression and activity, while KLF4 in turn modulates the phenotype and function of TME‐resident cells, thereby orchestrating pro or anti‐tumorigenic programs. Such bidirectional interactions could partly explain the dualistic nature of KLF4 in different cancer types. The recognition of this dynamic interplay has therapeutic implications. Modulating KLF4 activity—whether by enhancing tumor‐suppressive functions or dampening oncogenic signaling—could reprogram the TME toward an anti‐tumor state. This concept aligns with the growing interest in therapies targeting specific cellular or molecular components of the TME or interfering with its protumorigenic signaling networks [10]. In this review, we focus specifically on the immune and ECM compartments of the TME, as accumulating evidence indicates that these are the domains where KLF4 exerts the most direct and mechanistically well‐characterized effects [11]. We summarize how differential KLF4 expression in various TME cell types impacts immune regulation, ECM remodeling, and tumor progression, and discuss the therapeutic potential and current challenges of strategies targeting KLF4–TME interactions.

## 2 Gene Expression and Protein Domain Structure of KLF4

The KLF4 gene promoter region is enriched in GC boxes and binding sites for Sp1/KLF family protein [12]. The promoter region, along with distal cis‐regulatory elements, integrates regulatory cues from diverse signaling pathways and transcription factors such as p53, NF‐κB, and STATs [13, 14, 15]. Epigenetic modifications, notably methylation of promoter CpG islands, silence KLF4 expression [16]. MicroRNAs (miRNAs) such as miR‐145 exert negative control by targeting the 3′UTR region of KLF4 mRNA, repressing its translation or accelerating mRNA degradation [17]. Long noncoding RNAs (lncRNAs) may exert fine‐tuned control over KLF4 expression by interacting with the KLF4 locus or recruiting chromatin‐modifying complexes [18]. Furthermore, post‐transcriptional regulatory mechanisms through circular RNA (circRNA) further fine‐tune the ultimate expression levels of KLF4 protein [19].

**FIGURE 1:**
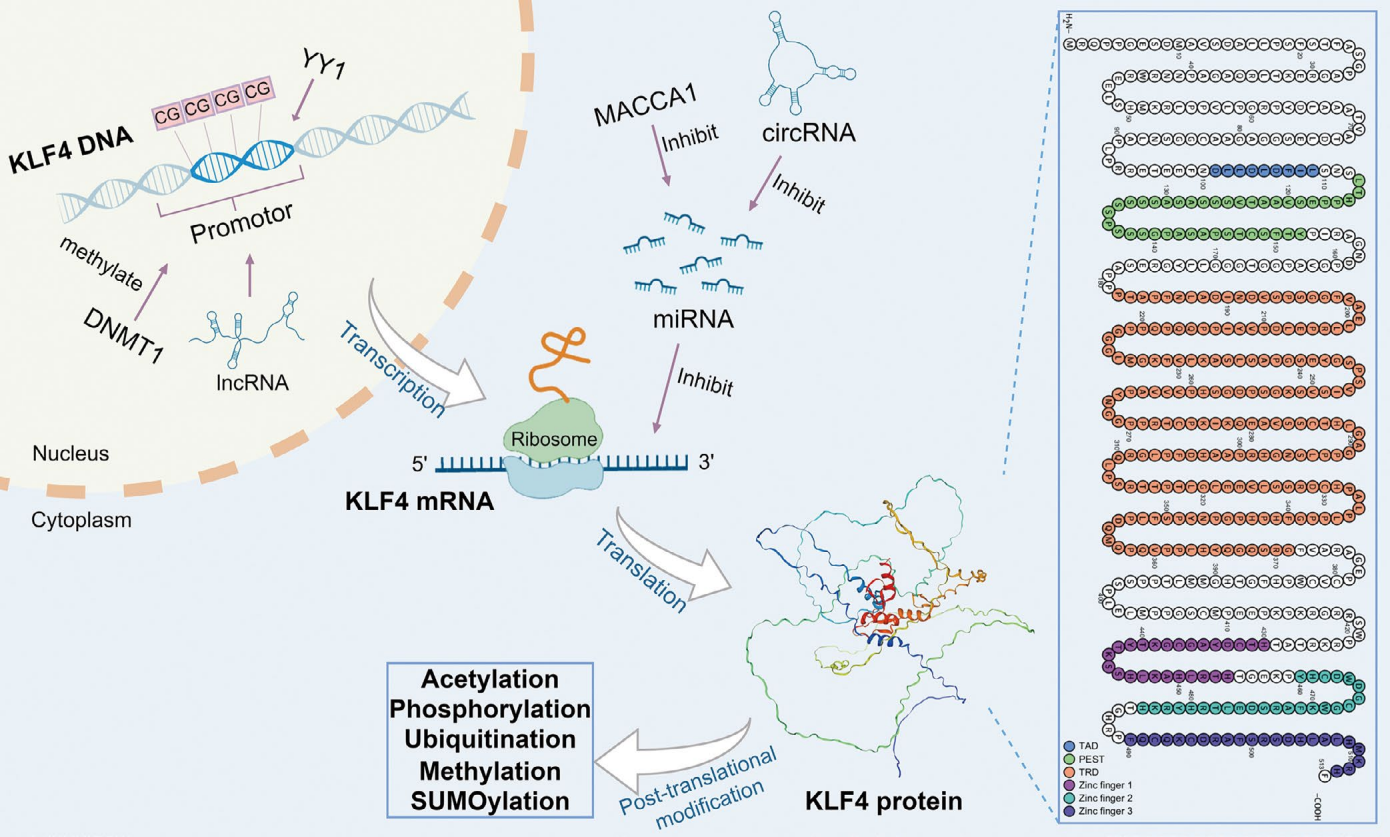
Gene expression and protein domain structure of KLF4. The expression of the KLF4 gene is regulated by multiple factors. Within the nucleus, DNA methylation (at CG sites) and lncRNAs participate in the transcriptional regulation process. The YY1 transcription factor binds to the promoter region of the KLF4 gene, promoting its transcription. The transcribed KLF4 mRNA is exported from the nucleus to the cytoplasm, where it is translated into KLF4 protein on ribosomes. The stability of KLF4 mRNA is subject to inhibitory regulation by miRNAs, which bind to KLF4 mRNA, inhibiting its translation or promoting its degradation, thereby negatively regulating KLF4 protein expression levels. However, the circRNAs and MACCA1 could suppress and degrade the miRNAs and ultimately upregulate the expression of KLF4. The newly synthesized KLF4 protein can undergo various post‐translational modifications, including acetylation, phosphorylation, ubiquitination, methylation, and SUMOylation. These modifications can affect the stability and function of the KLF4 protein. The human KLF4 protein contains several functional domains, including: a transcriptional activation domain (TAD, amino acids 101–109), a PEST domain rich in proline, glutamic acid, serine and threonine (amino acids 113–152), and a threonine‐ and arginine‐rich transcriptional repression domain (TRD, amino acids 181–371). The C‐terminus of the protein contains three zinc finger domains (Zinc finger 1–3, amino acids 430–454, 460–484, 490–512), which mediate KLF4 binding to target DNA.

The functional versatility of the KLF4 protein is not solely attributed to the intricate regulation of its gene expression but is also linked to its modular protein domain architecture [5]. The N‐terminal region constitutes its transcriptional activation and repression domain, which mediates protein–protein interactions with transcriptional coactivators or corepressors, thereby directing KLF4‐mediated positive or negative regulation of target gene transcription [20]. Two NLS sequences ensure efficient nuclear import of KLF4 protein, enabling its localization to chromatin. The C‐terminal zinc finger domain, composed of three tandem C2H2‐type zinc fingers, serves as the core structural element for KLF4's DNA recognition and binding. The linker peptides between zinc finger domains also contribute to the regulation of DNA‐binding specificity and affinity [5]. Besides, KLF4 protein harbors post‐translational modification sites, including acetylation, phosphorylation, ubiquitination and SUMOylation, which dynamically regulate KLF4 protein stability, subcellular localization, and transcriptional activity [21, 22, 23]. The basic gene expression and protein domain structure of KLF4 are shown in Figure 1.

## 3 The Unique Roles of KLF4 in TME

The TME is a dynamic and heterogeneous biological network that results from the interaction of cancer cells with infiltrating immune cells, stromal cells, blood vessels, the ECM, secretory products and specific environmental conditions [24]. The TME can be subdivided into nine specialized microenvironments, namely, hypoxic niche, immune microenvironment, metabolism microenvironment, acidic niche, innervated niche, cancer stem cell (CSC) niche, mechanical microenvironment, matrix microenvironment, and microbial microenvironment, which seem to be better targets for cancer treatment compared to the whole TME [25, 26]. Here, we place particular emphasis on the CSC niche, immune and matrix microenvironments because these compartments currently have the most direct and consistent evidence linking KLF4 to their modulation [8, 27, 28, 29].

**FIGURE 2:**
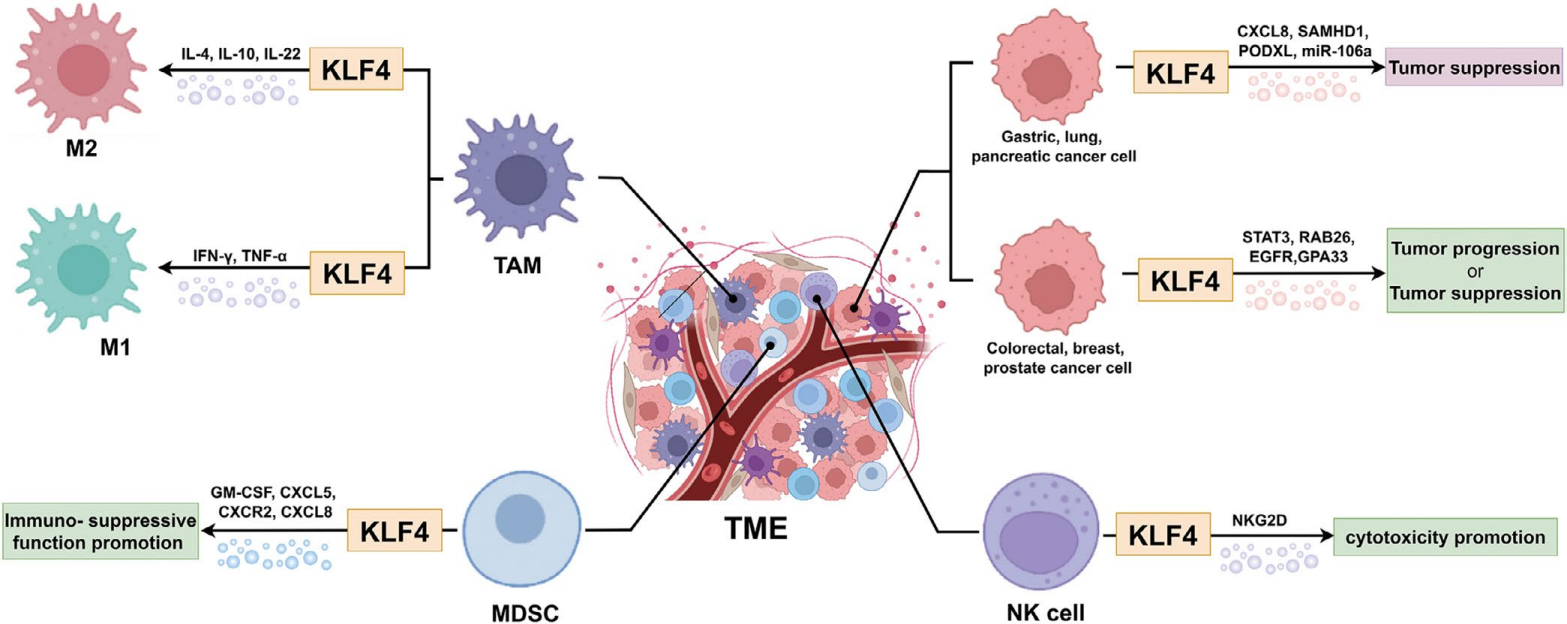
KLF4 regulates the function of cancer cells and immune cells in the TME. Cancer Cells: KLF4 exhibits diverse roles in different cancer cell types. In gastric, lung, and pancreatic cancer cells, KLF4 is primarily associated with tumor suppression, possibly mediated by the CXCL8, SAMHD1, PODXL, miR‐106a, and so on. Nevertheless, in colorectal, breast, and prostate cancer cells, KLF4 can function in either tumor progression or tumor suppression, potentially through mechanisms involving STAT3, RAB26, EGFR, GPA33, and so on. TAM: KLF4 influences macrophage polarization. KLF4 promotes macrophage polarization toward the M2 phenotype, potentially involving cytokines such as IL‐4, IL‐10, and IL‐22. M2 macrophages are generally associated with tumor promotion. KLF4 also promotes macrophage polarization toward the M1 phenotype, potentially involving cytokines such as IFN‐γ and TNF‐α. M1 macrophages typically exhibit anti‐tumor activity. MDSCs: KLF4 promotes the immunosuppressive function of MDSCs, potentially related to cytokines and chemokines such as GM‐CSF, CXCL5, CXCR2, and CXCL8. NK Cells: KLF4 enhances NK cell‐mediated cytotoxicity against cancer cells, possibly through the NKG2D receptor pathway.

KLF4 is recognized as a pivotal “bridge” molecule linking the immune system and tumor immunity [30]. By integrating its DNA‐binding ability with the selective recruitment of transcriptional coregulators and by undergoing fine‐tuned post‐translational modifications, KLF4 exerts precise control over the differentiation and polarization of diverse immune cell subsets [11]. For example, in macrophages, KLF4 drives M1/M2 polarization via cooperation with STAT6 and suppression of NF‐κB signaling, thereby shaping the landscape of tumor immune evasion [31, 32]. Beyond myeloid regulation, KLF4 directly influences T helper 17 (Th17) lineage commitment and modulates CD8^+^ T cell proliferation, memory development, and exhaustion dynamics [33]. Furthermore, elevated KLF4 expression exerts context‐dependent effects, either facilitating or limiting the infiltration of immune cells into tumor tissue [34]. Collectively, KLF4 serves as a central node in both innate and adaptive immunity in cancer settings. Moreover, as one of the Yamanaka factors, KLF4 is deeply connected with the CSC niche and could act as the seed for the proliferation and metastasis of cancer cells. Besides, KLF4 also functions importantly in regulating the matrix microenvironment. Genetic activation of KLF4 in perivascular cells can enhance ECM production and establish a prometastatic fibronectin‐rich environment [28]. Other TME components are not extensively discussed here because current literature provides limited or less direct evidence for a specific regulatory role of KLF4 in these contexts; however, they remain important avenues for future investigation. The following discussion will focus on how KLF4 expression influences the abundance or activity of specific cell types within the TME, and how these changes, in turn, affect tumor progression (Figure 2).

## 4 KLF4: The Cancer Cell Chameleon—Friend, Foe, or Both

The function of KLF4 in tumorigenesis is multifaceted, affecting apoptosis, proliferation, differentiation, and invasion of cancer cells [35]. The communication between KLF4 and cancer cells involves the regulation of diverse genes and biological factors by directly binding to their gene promoters or the downstream effectors. Among its key downstream targets, P21, P27, P53, and Cyclin‐D1 are considered particularly significant [5]. Evidence indicates that the role of KLF4 in cancer cells is dualistic, determined by the cancer type or subtype and the specific molecular regulation within signaling pathways [36, 37]. Even within the same cancer types, KLF4 can exhibit contradictory effects, further emphasizing the complexity of its involvement in tumor biology [38, 39]. Overall, however, KLF4 tends to act as a tumor suppressor, functioning as a key anti‐cancer force.

### 4.1 KLF4: The Cancer Fighter With a Few Secrets up Its Sleeve

In GC, KLF4 is consistently characterized as a tumor suppressor. The promoter region of the KLF4 gene is highly methylated in GC tissues, which is often exacerbated by *Helicobacter pylori* and results in markedly reduced expression levels through Cag pathogenicity island protein A (CagA) gene transduction [6, 40, 41]. In addition to the aforementioned gene methylation, microRNAs such as miR‐103 and miR‐135b‐5p can bind to the 3′ untranslated region (3′UTR) of KLF4 mRNA, thereby inhibiting its translation and promoting its degradation, ultimately resulting in downregulation of KLF4 protein expression [42, 43]. A cohort of downstream targets regulated by KLF4 has also been reported, such as Smad7 and SAMHD1 [44, 45]. In 2024, Y. Zang et al. reported that in GC, KLF4 transcription is downregulated due to STAT5A binding to its promoter region. The upregulation of STAT5A results from METTL3‐mediated m^6^A modification, which enhances the stability of STAT5A mRNA, thereby indirectly reducing KLF4 expression [46]. These insights could pave the way for potential therapeutic strategies for GC.

Similarly, in lung cancer cells, KLF4 is also considered to possess tumor‐suppressive capabilities [34, 47, 48]. KLF4 attenuates the invasiveness of lung cancer cells by transcriptionally downregulating NF‐κB2 and CXCR2, thereby attenuating prometastatic signaling pathways. Concurrently, it enhances the infiltration of CD4^+^ and CD8^+^ T cells as well as macrophages into the TME. These findings suggest that the impact of KLF4 extends beyond the modulation of individual cancer cell behavior, playing a broader role in shaping the tumor ecosystem [34]. In 2024, Zhuoshi Li et al. found that MACC1 inhibits the expression of miR‐25, thereby delaying the degradation of KLF4 mRNA and stabilizing KLF4, which suppresses the dedifferentiation process of non‐CSCs in lung cancer [49]. In colorectal cancer (CRC), KLF4's role transcends simple growth inhibition, acting as a regulator of cellular homeostasis [38, 50]. In 2025, Chunfei Li et al. reported that KLF4 suppresses CRC tumorigenesis by transcriptionally activating RING finger protein 112, which in turn promotes the ubiquitination and proteasomal degradation of the oncoprotein N‐α‐acetyltransferase 40 [51]. Similarly, in pancreatic cancer, KLF4 also exhibits a relatively consistent tumor‐suppressing effect [17, 52].

In addition to inhibiting the self‐proliferation and other functions of cancer cells, KLF4 can also enhance the sensitivity of cancer cells to chemotherapeutic drugs and radiotherapy, thereby improving treatment efficacy [53, 54]. In 2025, Ling Ma et al. showed that KLF4 can activate LATS2 and mTORC1 signaling to induce DNA damage in ovarian cancer cells and consequently increase their sensitivity to cisplatin [55, 56]. This suggests that combining KLF4 targeted therapy with conventional chemo‐ and radiotherapy may enhance therapeutic efficacy and reduce recurrence.

### 4.2 The Oncogenic Roles of KLF4: Fueling Cancer Growth and Resistance

While initially characterized as a tumor suppressor, the functional landscape of KLF4 is far more nuanced. Even within a single cancer type, such as breast, prostate cancer and CRC, KLF4 can exhibit opposing activities, acting as both a suppressor and promoter, a duality dictated by its expression level and the intricate TME [7, 8, 14, 18, 51, 57]. For instance, in prostate cancer, miR‐7, miR‐148‐3p, and miR‐152‐3p function as upstream regulators that downregulate KLF4 expression, thereby inhibiting cancer cell proliferation [58, 59]. Furthermore, the role of KLF4 in rarer malignancies like Wilms' tumor and melanoma through circRNAs and miRNAs highlights its capacity to drive tumorigenesis in specific contexts [60, 61]. Specifically, in melanoma, miR‐150‐3p downregulates KLF4 expression, thereby preventing KLF4 from binding to the promoter region of NCK2 and suppressing its transcription, ultimately leading to inhibition of tumor cell growth. This functional plasticity underscores the importance of considering the cellular and environmental background when interpreting KLF4's role in cancer.

A key to understanding this duality lies in KLF4's well‐established function as a regulator of cellular stemness, a property intrinsically linked to CSCs. In fact, KLF4 is widely acknowledged as a definitive marker of CSCs. Inhibition of KLF4 could suppress CSCs self‐renewal and cell migration, impeding tumor growth and metastasis [8, 38, 62, 63, 64]. MiRNA pathways, including miR‐29a, miR‐7‐5p, and miR‐48, are increasingly recognized as critical mediators of KLF4's link with CSCs [8, 38, 62]. Furthermore, interactions with proteins like integrin β4, Secreted Mucin 5 ACand EphA2 further refine KLF4's intricate control over stemness‐related signaling in the TME [63, 64, 65]. Deciphering the precise molecular choreography of KLF4 in CSCs is crucial, as it may unveil novel therapeutic vulnerabilities. Table 1 summarizes the signaling pathways modulated by KLF4 in the above types of cancer cells.

**TABLE 1:** The signaling pathways modulated by KLF4 in the above types of cancer cells.

Cancer type	Role of KLF4 on cancer cells	Molecules regulated by KLF4	Molecules regulate KLF4
Gastric cancer	Suppress	CXCL8 [41] miR‐106a [44] PODXL [6] SAMHD1 [45] TGF‐β, Notch, and Wnt signaling pathways [66]	CagA/TET1 [40] SNHG5/miR‐32 [67] miR‐103 [42] LINC00673 [68] miR‐135b‐5p [43]
Lung cancer	Suppress	OTUD1 [47] TIMP3 [22] SPARC [69] PLAC8 [70]	miR‐25 [71] LINC00852/hsa‐miR‐145‐5p [72] TRHDE‐AS1/miR‐103 [73] USP10 [22] miR‐3120‐5p [74] PTEN [75] HDAC [76]
Pancreatic cancer	Suppress	LDHA [77] CD44 [78] NAG‐1, p21 [79] MSI2 [80] GPRC5A [17]	MCC‐555 [79] miR‐135b‐5p [17]
Colorectal cancer	Promote	NDRG2 [81] p21 [82] STAT3 [14] RAB26 [39] GPA33 [83]	TUG1, EZH2 [84] TCFL5_E8 [85] miR‐543 [86] Orlistat [87] PPARgamma [83]
	Suppress	MMP2 [88] miR‐153‐1 [84] μ‐protocadherin [89] Bmi1 [90] IFITM3 [91] PiHL/EZH2/HMGA2 [92] RNF112 [51] RAB26 [39] HMGB1, hTERT [93]	miR‐29a [88] miR‐92a [50] TUG1, EZH2 [84] miR‐7‐5p [38] miR‐103/(and)107 [94] E2F3/MEX3A [95] miR‐206 [96]
Breast cancer	Promote	S100A14 [97] PFKP [98] Notch signaling [99]	TPA [97] MED27 [100] ATXN3 [101] FBXO32 [102] EGR1 [36] miR‐484 [62] miR‐7 [103] miR‐29a [8] DYRK2/AR [104]
	Suppress	MMP2 [19] EGFR [7] Eralpha [105] MAPK signaling pathway [106] E‐Cadherin [107]	circEHMT1/miR‐1233‐3p [19] DIM [16] AGPAT9 [108] Cy3G [109] DDX3X [110] TNF‐α [111]
Prostate cancer	Promote	miR‐7 [112] ARPC5/ADAM17 [57] PI3K/Akt/p21 [58] KRT6, KRT13 [113]	miR‐148‐3p, miR‐152‐3p [59] UCA1 [113]
	Suppress	AKT/p21 signal pathway [58] SLUG [114]	miR‐32‐5p [115] LINC00673 [18]

## 5 KLF4: The Traffic Cop for Macrophage Polarization–M1 or M2

Tumor‐associated macrophages (TAMs) are vital components of the TME, infiltrating tumor masses and playing crucial roles in cancer progression. They are predominantly classified into two subtypes: the M1 type, which is activated through the classical pathway and is anti‐tumor in nature, and the M2 type, activated by alternative pathways and known for its protumor characteristics. The M2 phenotype is significantly correlated with the progression of cancer and unfavorable outcomes [116].

### 5.1 KLF4's Role in Macrophage Activation and Differentiation

A decade ago, seminal work identified that KLF4 is a pivotal regulator of macrophage activation, with its interaction with the NF‐κB family member p65 that cooperatively induces the activation of the iNOS promoter, a hallmark of activated macrophages, and at the same time inhibiting the anti‐inflammatory TGF‐β1/Smad3 signaling pathway through competitive interaction with Smad3 for binding to the C‐terminal domain of the coactivator p300/CBP [117, 118]. As research has delved deeper, it has elucidated the intricate connection between the molecular structure of KLF4 and the differentiation of macrophages. Wild‐type KLF4 induces terminal macrophage differentiation. Nonetheless, KLF4 mutants with structural aberrations in C‐terminal zinc finger domains have been shown to stimulate self‐renewal and arrest the maturation of macrophages. The disruption of KLF4's binding to its target DNA may lead to monocytic leukemia [117].

### 5.2 KLF4: Primarily Driving M2 Macrophage Polarization

Significant involvement of KLF4 in macrophage infiltration and polarization has been verified. KLF4 has been demonstrated to bidirectionally influence the polarization of macrophages through multiple signaling pathways, such as regulating the expression levels of pro‐inflammatory factors like IFN‐γ, LPS, TNF‐α and anti‐inflammatory factors including TGF‐β1, IL‐4, and IL‐10 [119, 120, 121, 122, 123]. However, KLF4 was generally found to be mainly implicated in the M2 polarization of macrophages, which, in turn, promotes tumorigenesis [123].

KLF4's role in TAM polarization is recognized as a process mediated by diverse signaling cascades. In HCC, KLF4 plays a pivotal role in TAMs by mediating Hedgehog (Hh) signaling–dependent M2 polarization and its associated immunosuppressive functions. Specifically, the downstream transcription factor Gli1 translocates into the nucleus, binds to the KLF4 promoter, and directly upregulates KLF4 expression. This activation drives the transcription of M2‐associated genes, such as arginase‐1, CD206, IL‐10, and TGF‐β1, while concurrently suppressing the expression of M1‐associated genes, including TNF‐α, iNOS, and IL‐6 [124]. Da‐Liang Ou et al. further elucidated that activated p38 MAPK promotes Creb1 phosphorylation. The activated Creb1 then binds to the KLF4 promoter, consequently enhancing the transcription of M2‐related genes [125]. Similarly, in glioblastoma, tumor‐derived kynurenine activates the AHR/KLF4/SOCS2/TRAF6 axis in TAMs, driving the expression of M2‐associated genes [29]. Gradually, the mechanism underlying KLF4 and miRNAs in TAM has been unraveled. The miR‐206 and miR‐29a could directly target and downregulate KLF4 by preventing the ribosome from translating the KLF4 mRNA into protein, and miR‐34a‐5p could accelerate KLF4 degradation, thereby steering TAM from the M2 phenotype to the M1 phenotype [29, 126, 127]. Further studies have extended to lncRNAs as upstream regulators of miRNAs. LncRNA HCG18, by reducing miR‐875‐3p levels within TAMs, consequently leads to an upregulation of KLF4 expression, thereby driving M2 polarization and alleviating GC progression [128]. In 2025, the transcriptional coactivator steroid receptor coactivator‐3 protein was found to promote the release of KLF4 in CRC cells, thereby facilitating the M2 polarization of TAMs [129].

Conversely, while often associated with M2 polarization or tumor‐promoting effects, increased KLF4 expression can, in certain contexts, promote M1 polarization. This can occur through post‐translational modifications. Small ubiquitin‐like modifier (SUMO)‐specific peptidase 1 (SENP1), functioning as the specific de‐SUMOylating enzyme for KLF4, increases KLF4 protein stability and enhances its transcriptional activity. This, in turn, promotes the polarization of TAMs to an M1 phenotype through regulation of the NF‐κB signaling pathway [23].

**FIGURE 3:**
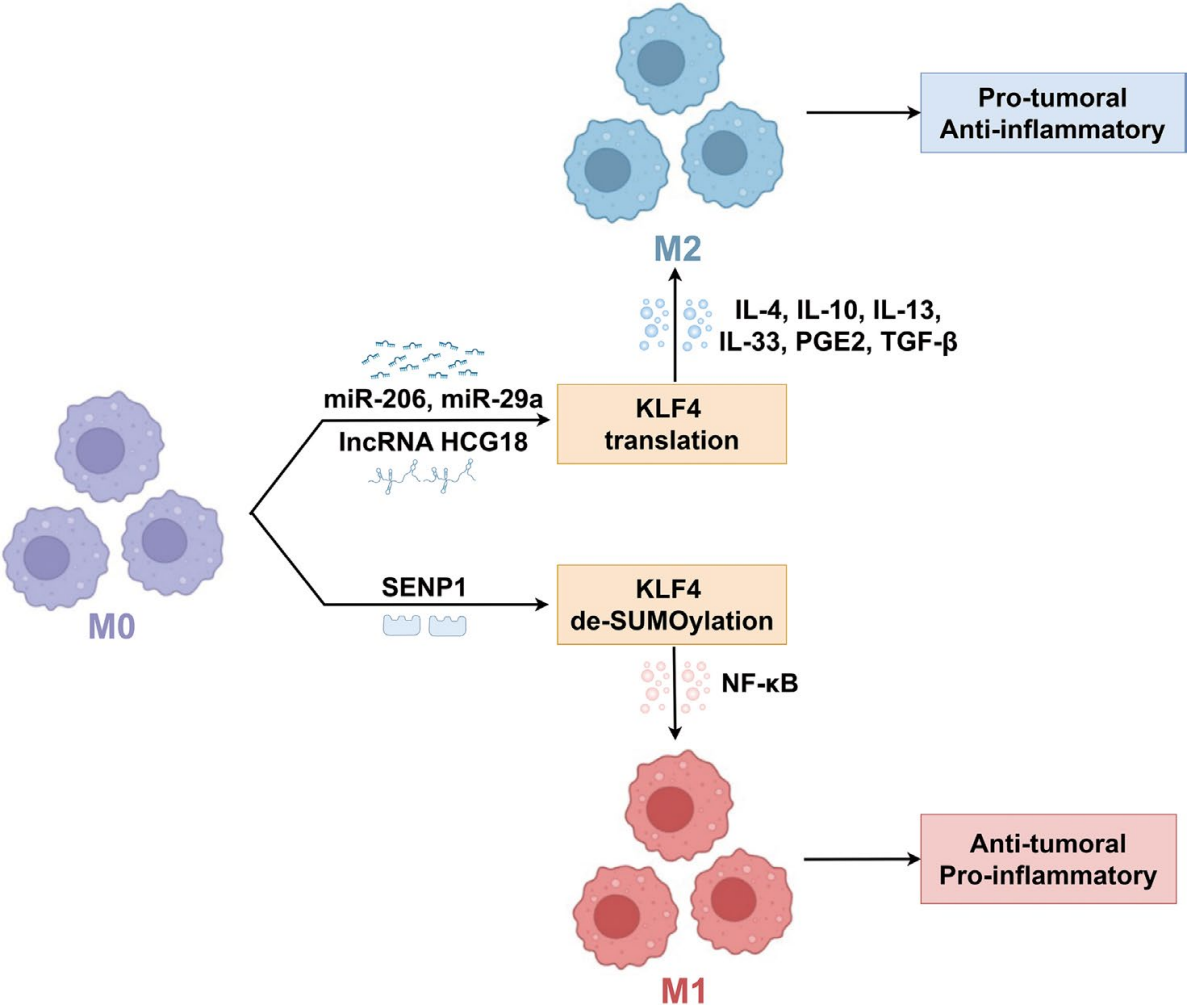
The role of KLF4 in TAM polarization. M0 to M2 Macrophage Polarization: In M0 macrophages, KLF4 mRNA expression is negatively regulated by miRNAs (miR‐206, miR‐29a) and lncRNA HCG18. Inhibition of the miRNAs depicted in the figure could upregulate KLF4 mRNA expression, leading to increased translation of KLF4 protein and subsequent upregulation of cytokines such as IL‐4, IL‐10, IL‐13, IL‐33, PGE2, and TGF‐β. The cascade ultimately promotes the polarization of M0 macrophages toward the M2 phenotype, which is characterized by a protumoral and anti‐inflammatory function. M0 to M1 Macrophage Polarization: Alternatively, SENP1 promotes the de‐SUMOylation of KLF4 protein in M0 macrophages. The de‐SUMOylation, in conjunction with NF‐κB signaling, leads to increased levels of KLF4 protein and drives the polarization of M0 macrophages toward the M1 phenotype, which exhibits anti‐tumoral and pro‐inflammatory properties.

These findings highlight KLF4 as a critical regulator of key signaling pathways that control TAM activities. The balance of a variety of pro and anti‐inflammatory signals determines the outcome of the interaction (Figure 3). Future research directions should prioritize targeting KLF4 to reprogram TAMs from a tumor‐promoting M2 phenotype to a tumor‐suppressing M1 phenotype, which could pave the way for novel cancer treatments [130].

## 6 How KLF4 Shapes T Cell Function and Fights Leukemia

In the TME, tumor‐infiltrating T lymphocytes exert a pivotal role in orchestrating the overall immune response and directly killing cancer cells. CD8^+^ T cells secrete large amounts of IFN‐γ and the protease granzyme B, which act synergistically to kill tumor cells [131]. Over the past decade, therapeutic strategies aimed at enhancing T cell activity have rapidly evolved [132, 133].

### 6.1 KLF4 Regulates T Cell Differentiation, Proliferation, and Exhaustion

In 2009, Takeshi Yamada et al. identified KLF4 as an inhibitor of naive CD8^+^ T cell proliferation, positing it downstream of T cell receptor signaling and activated by ELF4, thereby promoting cell cycle arrest [134]. Further evidence suggests that KLF4 downregulation is a prerequisite for T cell lineage commitment, as KLF4 overexpression severely impedes early T cell development, including thymocyte survival and TCRβ locus rearrangement [135]. Mechanistic studies have delved into the molecular details of KLF4 regulation. The E3 ligase Mule mediates KLF4 ubiquitination, promoting T cell entry into the S phase, suggesting that KLF4 degradation is a necessary step for cell cycle progression. Conversely, aberrant accumulation of KLF4 drives the expression of cell cycle inhibitors p21 and p27, as well as E2F2, subsequently affecting T cell proliferation [136].

Notably, KLF4's function extends beyond cell cycle regulation, playing a crucial role in T cell exhaustion [33]. KLF4‐expressing CD8^+^ T cells exhibit a phenotype of transient effector cells. Forced expression of KLF4 can promote the differentiation of CD8^+^ T cells toward the transient effector subset, enhance their anti‐tumor effector functions, and possess the potential to reverse CD8^+^ T cell exhaustion [33]. By modulating KLF4 expression or activity, there is hope to reshape the anti‐tumor immune function of T cells.

### 6.2 KLF4 as a Tumor Suppressor and Therapeutic Target in T‐ALL

During the last decade, significant progress has been made in understanding the role of KLF4 in the pathogenesis of T‐cell acute lymphoblastic leukemia (T‐ALL). In T‐ALL, KLF4 acts as a tumor suppressor. Mutations in the 3′ untranslated region (UTR) and the zinc‐finger domain of the KLF4 gene lead to the loss of its tumor‐suppressing function, which may be a significant factor causing the progression of T‐ALL [137]. KLF4 can also induce T‐ALL cell apoptosis through the BCL2/BCLXL pathway, along with inhibiting T cell‐associated genes including NOTCH1, BCL11B, GATA3, and TCF7, which result in the suppression of T‐ALL progression [138]. Subsequently, the pivotal role of the KLF4‐MAP2K7 pathway in the pathogenesis of T‐ALL was discovered. KLF4 gene silencing in T‐ALL will lead to aberrant activation of the mitogen‐activated protein kinase MAP2K7 and the downstream effectors c‐Jun NH(2)‐terminal kinase (JNK) and activating transcription factor 2 (ATF2) pathway, thus promoting the expansion of leukemia cells. These studies provide proof‐of‐principle preclinical data supporting KLF4/MAP2K7/JNK/ATF2 pathway inhibition as a potential targeted therapy for T‐ALL [139, 140].

The in‐depth investigation discovered a small molecule, 5Z‐7‐oxozeaenol (5Z7O), capable of targeting the aforementioned KLF4/MAP2K7 pathway. The study demonstrated that the combination of 5Z7O and dexamethasone exhibits synergistic cytotoxicity, offering a foundation for prospective preclinical research [141]. In 2024, Mina Noura et al. found that the small molecule inducer of KLF4, APTO‐253, exerts its anti‐leukemic effects in T‐ALL cells by targeting super‐enhancer (SE)‐driven expression of TAL1, a gene frequently disrupted in T‐ALL. Later, KLF4 was discovered to induce T‐cell differentiation and apoptosis in T‐ALL cells carrying activating mutations in NOTCH3. Specifically, treatment with APTO‐253 upregulated KLF4 expression, which directly bound to the NOTCH3 promoter and suppressed NOTCH3 transcription. This downregulation facilitated the differentiation of CD4/CD8 double‐positive cells into CD4 single‐positive cells, followed by apoptosis, thereby effectively inhibiting the proliferation of T‐ALL cells [142]. These result indicates that APT0‐253 holds promise as a therapeutic agent for targeting oncogenic signaling in T‐ALL cells. Collectively, KLF4 is a potential strategy for T‐ALL and may represent novel therapeutic approaches for patients with poor prognosis [143].

## 7 KLF4: Helping B Cells Grow or Holding Them Back

B cells play a dual role in the TME. They can produce lymphotoxin to promote tumor growth and angiogenesis and antibodies against tumor‐specific antigens to foster anti‐tumor immune responses. Indirect evidence suggests that KLF4 might participate in regulating B cell fate and function by influencing key transcription factors (e.g., Pax5, IRF4), BCR signaling, and cellular metabolism [144, 145]. However, no direct evidence demonstrates that KLF4 regulates the expression of the specific markers of the pro or anti‐tumorigenic B cell subsets.

### 7.1 Roles of KLF4 in B Cell Proliferation and Maturation

In the 2000s, a delay was observed in entering the S phase of the cell cycle in B cells and a reduction in the number of mature B cells in the absence of KLF4, which further demonstrated that KLF4 regulates their activation‐induced proliferation by directly acting on the promoter of cyclin D2, a cell cycle protein [146]. Paradoxically, studies a year later observed that forced expression of KLF4 in proliferating B cell blasts caused a G1 cell cycle arrest and indicated that KLF4 inhibits the proliferation of B cells [147]. The apparent discrepancy in findings could be attributed to the variable role of KLF4 across different phases of the cell cycle, potentially promoting the G1 to S phase transition while inhibiting the cell cycle's progression at other junctures [148].

Research also shed light on the critical role of KLF4 in plasma cell maturation, with implications for anti‐tumor immunity [149, 150]. Artificial upregulation of KLF4 in plasma blasts significantly promotes their maturation into both early and long‐lived plasma cells. This enhanced maturation is achieved, at least in part, by preventing cell apoptosis, potentially through suppressing p53 activity and thus reducing p53‐mediated apoptosis [149, 150]. Crucially, this KLF4‐driven plasma cell maturation directly translates to enhanced antibody production. Given the pivotal role of antibodies in anti‐tumor immunity, particularly in mediating cancer cell clearance and long‐term protection, these findings suggest that understanding and manipulating KLF4 signaling in plasma cells holds significant therapeutic potential for enhancing antibody‐mediated cancer immunotherapy.

### 7.2 KLF4 and B‐Cell Lymphoma: Tumor Suppression

In the 2010s, research has predominantly focused on exploring the role of KLF4 in B‐cell lymphoma [151]. KLF4 was identified as a tumor suppressor in B‐cell lymphoma. The epigenetic silencing of KLF4 in B‐cell lymphomas, particularly in classical Hodgkin's lymphoma (CHL), is thought to bolster lymphoma survival through the relaxation of cell‐cycle constraints and protection from apoptosis [152]. The KLF4 methylation in non‐Hodgkin's lymphoma (NHL) is also quite pronounced and unaffected by hypomethylating drugs [153, 154]. A specific transcription factor Yin‐Yang 1 (YY1) has been discovered to be coexpressed with KLF4 and to positively regulate the KLF4 expression, suggesting that both YY1 and KLF4 could serve as potential therapeutic biomarkers for NHL [155, 156]. The discovery of these regulatory molecules may pave the way for new therapeutic approaches to B‐cell lymphoma.

## 8 KLF4 Affects MDSC Chemotaxis

Myeloid‐derived suppressor cells (MDSCs) are a heterogeneous population of activated immature myeloid cells generated during many pathologic conditions, especially in cancer. MDSCs potently suppress T‐cell activity and thus contribute to the immune escape of malignant tumors [157]. Multiple chemokines, such as CXCL8 and CXCL5, bind to their cognate receptors expressed on the surface of MDSCs, activating downstream signaling pathways that guide MDSC migration to the TME [158, 159, 160].

Notably, mechanistic studies revealed the opposite effects of KLF4 on MDSC recruitment and immunosuppressive activity in breast cancer and GC. In breast cancer, the lack of strong pro‐inflammatory pathogenic stimulation renders KLF4 more likely to support tumor immune‐escape pathways. In breast cancer cells, KLF4 functions as an oncogenic factor, upregulating CXCL5 and subsequently inducing GM‐CSF expression in the bone marrow. CXCL5 not only promotes MDSC maintenance via GM‐CSF but also drives their recruitment into the tumor site through the CXCL5/CXCR2 axis, thereby enhancing immunosuppressive activity and tumor progression [161]. In contrast, in GC, persistent *Helicobacter pylori* infection, together with the activity of its virulence factor CagA, induces chronic inflammation, markedly upregulates CXCL8 expression, and simultaneously suppresses KLF4, creating a feed‐forward loop that favors MDSC accumulation and immune evasion. KLF4 acts as a tumor suppressor by directly binding to the CXCL8 promoter and repressing its transcription, leading to reduced CXCL8/CXCR1/2‐mediated MDSC chemotaxis [41]. Therefore, the net effect of KLF4 on MDSCs appears to be cancer‐type specific, largely dictated by the identity of the dominant chemokine downstream of KLF4 (CXCL5 vs. CXCL8), the upstream inflammatory signals within the TME, and the intrinsic role of KLF4 as either oncogene or tumor suppressor in the given tissue.

## 9 KLF4 Restores NK Cell Surveillance

Natural killer (NK) cells are powerful effectors of innate immunity that constitute a first line of defense against cancer and are promising targets for cellular immune therapy. However, NK cell therapy still faces challenges, such as insufficient NK cell infiltration, dysfunction, and metabolic dysregulation [162]. These challenges are largely attributed to the multiple immunosuppressive mechanisms present in the TME [26].

KLF4, as a pluripotency gene, promotes the survival and differentiation of NK cells. The absence of the KLF4 gene was observed to result in a significant reduction in NK cells [163]. Beyond its role in NK cell development and maintenance, KLF4 emerges as a critical orchestrator of NK cell‐mediated tumor immune surveillance, particularly through the immunoreceptor NKG2D, which is expressed on NK cells and subsets of T cells. Acute myeloid leukemia (AML) often evades this surveillance by downregulating NKG2D ligands (NKG2D‐L), especially MICA. In 2023, Reem Alkhayer et al. elucidated multiple mechanisms by which KLF4 regulates MICA expression and enhances NK cell anti‐leukemia activity. Regulated by the p300/CBP coactivator family, KLF4 directly binds and transactivates the MICA promoter, thereby increasing its expression and rendering AML cells more susceptible to NK cell recognition and cytotoxicity. KLF4 also links DNA damage signaling to MICA upregulation, integrating cellular stress responses into immune activation. The small molecule APTO‐253, a clinical‐stage agent, was shown to induce KLF4 and MICA expression in a dose‐dependent manner across multiple AML cell lines. AML cells treated with APTO‐253 not only exhibited elevated surface MICA but also triggered significantly higher secretion of pro‐inflammatory cytokines (IFN‐γ and TNF‐α) from cocultured NK cells. Furthermore, APTO‐253 upregulated additional NKG2D ligands, including MICB and ULBP1, suggesting a broader role for KLF4 in orchestrating the NKG2D‐L family. This direct link between APTO253‐driven KLF4/MICA induction and enhanced NK cell‐mediated clearance reveals a novel therapeutic axis, offering a promising strategy to restore anti‐tumor immunity in AML [164].

## 10 KLF4's Role in DCs and Neutrophils: Underexplored Area

In the TME, dendritic cells (DCs) often experience impaired cross‐presentation due to multiple inhibitory signals, causing them to favor immune tolerance over effective antitumor response [165]. The regulation of DC's function by KLF4 within the TME has been underexplored. However, in fundamental mechanistic research, the critical role of KLF4 in driving DC differentiation and development has been progressively clarified. DCs can be broadly classified into conventional DCs (cDCs) and plasmacytoid DCs (pDCs). cDCs are further divided into two major subsets, cDC1 and cDC2, with cDC2 comprising two distinct lineages: T‐bet^+^ DC2A and T‐bet^−^ DC2B. KLF4 is a transcription factor selectively expressed within the cDC lineage and serves as an actionable lineage demarcation marker, clearly segregating cDC and pDC lineages at the progenitor stage [166]. Most importantly, KLF4 directly governs the generation of CD115^+^ pre‐DC2s, the progenitors of DC2B, thereby maintaining DC2B abundance and antigen‐presenting capacity, while also promoting the differentiation of Siglec‐H^+^ pre‐DC2s, the progenitors of DC2A, into mature DC2A cells. Therefore, KLF4 finely orchestrates the heterogeneity of cDC2 subsets and their distinct developmental trajectories. Modulating KLF4 activity offers a potential strategy to alter the abundance and functional properties of DC2 subsets in cancer vaccines or immunotherapy, aiming to enhance antigen presentation [167].

Neutrophils differentiate into either anti‐tumorigenic N1 or protumorigenic N2 phenotypes in TME. Specifically, cytokines like TGF‐β and IL‐10 drive neutrophils toward the N2 phenotype, whereas IFN‐γ and GM‐CSF promote the development of the N1 phenotype [168]. Compared with TAM polarization, research on neutrophil polarization in the TME remains lacking, which is a largely untapped area, offering significant opportunities for future investigation. Existing literature primarily focuses on the role of KLF4 in neutrophils during inflammatory diseases, where increased KLF4 expression is generally associated with enhanced neutrophil activation, infiltration, and immune responses [169, 170, 171].

## 11 KLF4 In the ECM: Building or Breaking Down the Tumor's Playground

The ECM, a vital constituent of the TME, provides mechanical support for tumor cells and engages in multiple cellular communications [172]. KLF4 exhibits a context‐dependent role in regulating the ECM in TME. Secreted protein acidic and rich in cysteine (SPARC) is an ECM protein that contributes to tumor progression and metastasis. In lung cancer, Yanbin Zhou et al. demonstrated that KLF4 functions as a tumor suppressor, and its ectopic expression markedly downregulates SPARC gene expression, thereby inhibiting the invasion of lung cancer cells [69]. Laminin‐5 is another predominant ECM protein but is produced by mammary epithelial cells. In contrast, in breast cancer cells, KLF4 acts as an oncogene and activation of KLF4 upregulates Laminin‐5 expression, which in turn facilitates tumor metastasis [173]. Furthermore, altered KLF4 expression in pericytes also impacts the ECM. Under the influence of tumor‐secreted factors, KLF4 expression is upregulated in pericytes, which drives them toward a less differentiated state. This phenotypic switch is accompanied by the loss of conventional pericyte markers, enhanced proliferation and migration, and increased synthesis of ECM components, ultimately contributing to the establishment of a prometastatic microenvironment enriched in fibronectin [28].

Cancer‐associated fibroblasts (CAFs) are the main contributors to ECM stiffness and degradation, which support and promote tumorigenesis [174]. Compared to CAFs, the lower expression level of KLF4 in normal fibroblasts suggests that KLF4 might play a protective role in impeding the transformation of normal fibroblasts into CAFs, possibly through the miR‐92a‐3p/KLF4/cholesterol 25‐hydroxylase (CH25H) axis [50]. Specifically, LINC01915 functions as a molecular sponge for miR‐92a‐3p, competitively binding to it and thereby relieving the repression of miR‐92a‐3p on its target gene, KLF4. The consequent upregulation of KLF4 enhances the transcriptional activity of CH25H. Then, CH25H activates the TGF‐β/Smad signaling pathway, leading to increased synthesis of ECM components like collagen and fibronectin and enhanced matrix degradation. This molecular cascade ultimately promotes the trans‐differentiation of normal fibroblasts into CAFs and creates a favorable environment for tumor cell invasion and metastasis [50].

## 12 KLF4: The Hub Gate for Cell Crosstalk in TME

KLF4 operates within the TME as part of a complex, dynamic network. Its regulatory effects rarely remain confined to a single cellular or molecular component; rather, perturbations in KLF4 expression often propagate through the system, eliciting a cascade of downstream “butterfly effects.” A prototypical example is KLF4‐mediated control of macrophage polarization, which in turn reshapes T‐cell activation, chemotaxis, and suppression [175].

Mechanistically, after KLF4 mediates Hh‐dependent polarization of TAMs toward the M2 phenotype, it further consolidates immunosuppression by repressing TAM‐derived CXCL9 and CXCL10, thereby limiting the recruitment of CD8^+^ T cells into the TME [124]. Extending beyond the Hh pathway, AHR‐driven upregulation of KLF4 suppresses NF‐κB activation in TAMs and promotes M2 polarization; in turn, this induces expression of the ecto‐nucleotidase CD39, which cooperates with CD73 to generate adenosine and precipitate CD8^+^ T cell dysfunction [29]. In line with the findings mentioned above, Da‐Liang Ou and colleagues demonstrated that the Creb1/KLF4 axis not only drives M2 polarization but also dampens the proliferation and activation of T cells in coculture systems [125]. Furthermore, KLF4 secreted by highly invasive CRC cells reinforces this immunosuppressive circuitry by simultaneously skewing TAMs toward an M2 state and upregulating PD‐L1 on TAMs, thereby inhibiting cytotoxic T lymphocyte (CTL) function, fostering T‐cell exhaustion, and facilitating immune evasion [129]. Conversely, when KLF4 functions as a tumor‐suppressive factor within the TME, as exemplified in lung adenocarcinoma, it downregulates NF‐κB2 and CXCR2, concomitantly restrains tumor cell invasion, and enhances the infiltration of CD4^+^ and CD8^+^ T cells, macrophages, and other immune subsets [34]. A comparable pattern has been observed in HCC [27]. Specifically, miR‐206 targets and reduces KLF4 expression, thereby augmenting the production of M1‐associated markers, including CCL2, and promoting CCR2‐dependent recruitment of CTLs to the TME, ultimately impeding tumor initiation [126].

KLF4 also orchestrates additional immune constituents within the TME. For example, by regulating DCs, KLF4 is a key determinant of CD4^+^ T cell homeostasis [176]. As noted above, KLF4 is essential for DC differentiation toward the cDC2 lineage, and intriguingly, it further promotes the maturation of cDC2a subsets, thereby enhancing the priming efficiency of Th17 cells and tuning immune thresholds at barrier sites [167]. In addition, KLF4 bridges DC–NK cell crosstalk. Loss of KLF4 reduces the pool of pre‐cDC progenitors and increases apoptosis in NK cells [163]. Moreover, KLF4 links cancer cells to MDSCs: alterations in cancer‐cell KLF4 expression modulate the production of chemokines such as CXCL5, thereby influencing MDSC recruitment into the TME [161].

KLF4 can also coordinate interactions among TME components via the ECM as an intermediary. MDSCs display phenotypic plasticity and can differentiate into fibrocytes—a distinct cell type that serves as a precursor of protumorigenic myofibroblasts. KLF4 deficiency in MDSCs markedly reduces pulmonary metastasis of breast cancer and melanoma, which correlates with decreased numbers of MDSC‐derived fibrocyte‐like cells and myofibroblasts. Mechanistically, KLF4 directly binds to the promoter of fibroblast‐specific protein 1 (FSP‐1), upregulating its expression and driving the generation of fibrocytes from MDSCs. These findings suggest that targeting KLF4 or FSP‐1 could be a feasible strategy to prevent tumor metastasis [177]. In silico transcriptomic analyses in pancreatic cancer further indicate that CAFs engage in strong crosstalk with immune cells. Through the MIF signaling axis, KLF4‐expressing CAFs promote immunosuppression and tumor invasion [178]. Beyond the previously discussed miR‐92a‐3p/KLF4/CH25H axis that regulates CAF conversion, CH25H and its enzymatic product 25‐hydroxycholesterol activate liver X receptor signaling to upregulate immunosuppression‐associated genes [50]. This, in turn, enhances interactions between CAFs and immunosuppressive cells such as M2 macrophages and regulatory T cells, thereby reinforcing CAF‐mediated suppression of antitumor immunity [179, 180].

## 13 Discussion

Heterogeneity of the TME is a critical hallmark of tumor biology, with distinct TME patterns observed across different tumor subtypes [25]. The function of KLF4 is intricately linked to these TME variations. Notably, in colorectal, breast, and prostate cancers, KLF4 exhibits remarkable context‐dependent and dual roles, acting as either a tumor promoter or suppressor depending on the intricate network of signaling pathways [7, 8, 14, 18, 51, 57]. This complexity significantly impedes the design and implementation of KLF4‐targeted therapies and the acquisition of compelling preclinical evidence for their safety and efficacy. However, in stark contrast to the aforementioned tumor types, KLF4 is consistently recognized as a tumor suppressor in GC and T‐ALL, with relatively specific mechanisms of action, providing a relatively robust preclinical rationale for therapeutic strategies targeting KLF4 [45, 46]. Pursuing KLF4‐targeted drug development in GC and T‐ALL represents a promising translational potential. Even so, it is still imperative to emphasize that inappropriate modulation of KLF4 activity could lead to severe adverse effects, such as promoting tumor growth or impairing normal cellular function. It requires extreme caution.

Given the plasticity of TAMs, reprogramming their polarization toward an anti‐tumor response is a high‐profile area of research. Therefore, targeting KLF4 to favorably alter the phenotypic ratio of TAMs, characterized by a reduction in M2 macrophages and an increase in M1 macrophages, represents a promising therapeutic strategy to promote anti‐tumor immunity [130]. Blocking KLF4's pro‐M2 interactions could be pursued, for instance, by designing small‐molecule inhibitors or peptide mimetics to prevent KLF4 from binding to the promoter regions of M2‐associated genes, or by targeting upstream activators of KLF4 in the M2 pathway [181]. Furthermore, leveraging the biological properties of lncRNAs is another possibility [128]. High‐throughput screening may identify lncRNAs that promote KLF4‐mediated M1 polarization. Subsequently, synthetic lncRNAs or antisense oligonucleotides may be developed as potential therapeutics [182, 183].

Dysregulation of KLF4 exerts pleiotropic and context‐dependent effects on both cellular and acellular components of the TME, mediated through complex and incompletely understood molecular mechanisms. A comprehensive elucidation of these mechanisms is paramount for the rational design of effective KLF4‐targeted cancer therapeutics. Therefore, the complexity of KLF4's regulatory networks necessitates methodological rigor in experimental investigations. Studies relying solely on transcript or protein abundance may offer a superficial and potentially misleading understanding of KLF4's functional impact within the TME, underscoring the critical need for experimental designs that integrate multiomics approaches and functional assays [184]. Despite these challenges, the potential of KLF4 as a biomarker for tumor diagnosis and prognosis remains a compelling area of investigation.

The KLF4‐targeting regulatory compound APTO‐253 has been shown to bind to DNA, and treatment of cells with APTO‐253 leads to DNA damage and cell‐cycle arrest at the G0–G1 phase, thereby inducing apoptosis [185]. In preclinical studies, APTO‐253 demonstrated potent activity against various leukemia cell lines and solid tumor cell lines, with IC_50_ values ranging from approximately 0.04 to 2.6 μM [186]. Moreover, treatment of ovarian cancer cells with APTO‐253 enhanced the therapeutic efficacy of paclitaxel and cisplatin [187]. In a study on triple‐negative breast cancer (TNBC), APTO‐253 also exhibited tumor‐suppressive effects. Specifically, NOXA, a proapoptotic member of the BCL‐2 family, was induced by APTO‐253 treatment in TNBC cells, promoting NOXA‐mediated apoptosis [188]. Further investigation in AML revealed that APTO‐253 upregulates CDKN1A (p21) expression, while downregulating MYC expression in a concentration‐ and time‐dependent manner [189].

In xenograft studies using intravenous administration of APTO‐253, significant antitumor activity was observed in mouse models bearing human HT‐29 colon adenocarcinoma, H460 nonsmall cell lung carcinoma, H226 squamous cell carcinoma/mesothelioma, and KG1 AML xenografts, without evidence of bone marrow suppression [185, 186, 190]. In addition, APTO‐253 was evaluated in vitro for its cytotoxic activity against 177 freshly isolated bone marrow samples from patients, including 80 cases of AML, 72 cases of chronic lymphocytic leukemia (CLL), and 25 cases of myelodysplasia/myeloproliferative neoplasms (MDS/MPN). The results demonstrated that AML samples were the most sensitive to APTO‐253, with 43 out of 80 samples (54%) exhibiting IC_50_ values below 1 μM [191].

**FIGURE 4:**
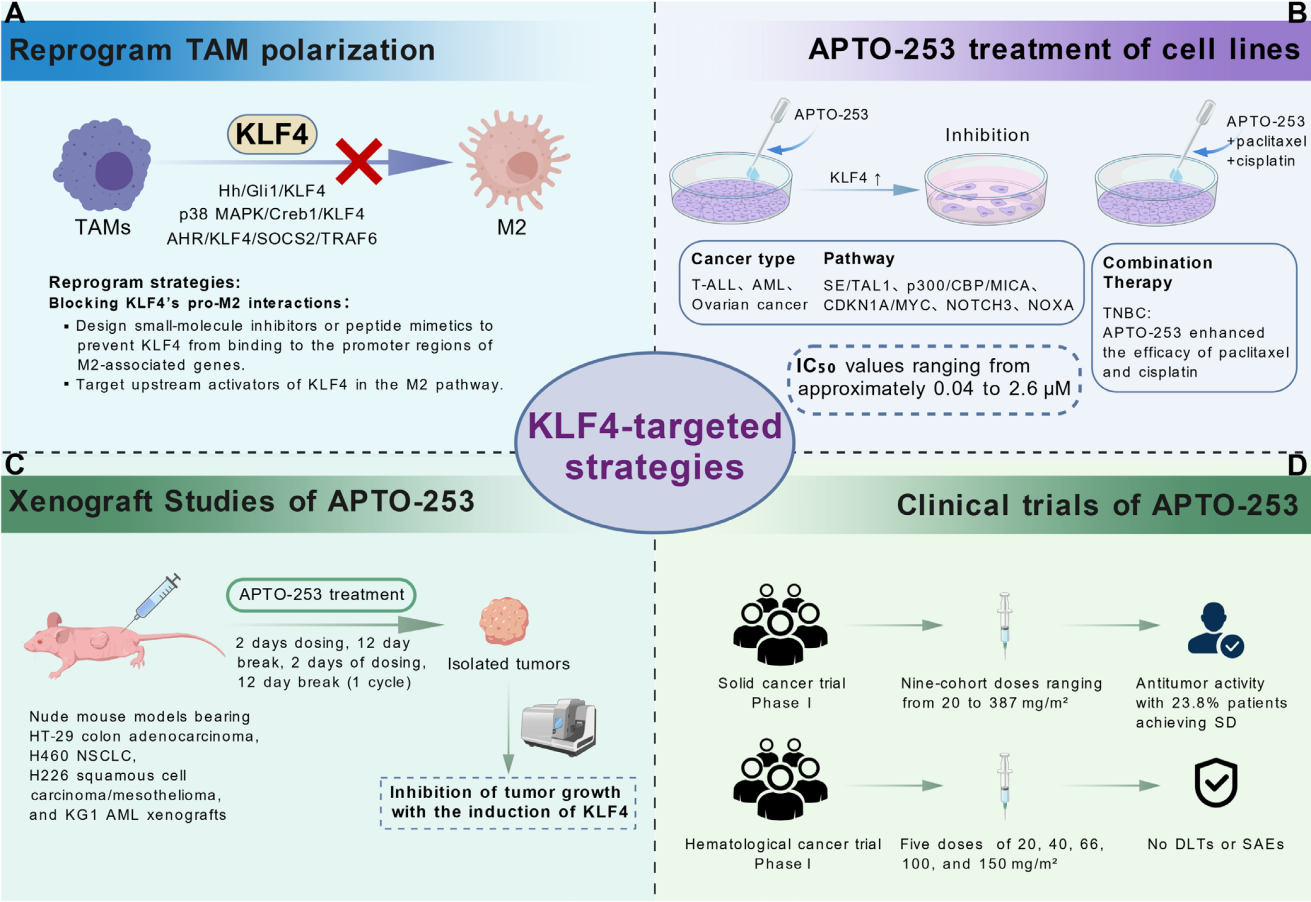
KLF4‐targeted therapeutic strategies in TME. (A) Reprogramming TAM polarization by blocking KLF4‐mediated M2 differentiation, via inhibition of upstream pathways (Hh/Gli1, p38 MAPK/Creb1, AHR/SOCS2/TRAF6) or disrupting KLF4 binding to promoters of M2‐associated genes. (B) APTO‐253 treatment in cancer cell lines (T‐ALL, AML, ovarian cancer, and TNBC) inhibits KLF4‐driven oncogenic pathways (SE/TAL1, p300/CBP/MICA, CDKN1A/MYC, NOTCH3, and NOXA), with IC50 values between 0.04 and 2.6 μM; combination therapy with paclitaxel and cisplatin enhances cytotoxicity. (C) Xenograft studies in nude mice bearing various tumors (colon adenocarcinoma, NSCLC, squamous cell carcinoma, mesothelioma, and AML) demonstrate that cyclical APTO‐253 dosing induces KLF4 expression and suppresses tumor growth. (D) Phase I clinical trials of APTO‐253 in solid and hematologic malignancies show antitumor activity (23.8% stable disease) across multiple dosing regimens without DLTs or SAEs.

The clinical trials of APTO‐253 have yielded initial signals of clinical benefit in Phase I trials for solid tumors (ClinicalTrials.gov registry number: NCT123226) and hematological malignancies (ClinicalTrials.gov registry number: NCT02267863), demonstrating tumor growth inhibition and favorable pharmacokinetic properties [192, 193]. In the solid tumor trial, a total of 32 patients were enrolled in a nine‐cohort dose‐escalation study, with doses ranging from 20 mg/m^2^ to 387 mg/m^2^. Despite the implementation of prophylactic measures, hypersensitivity reactions and transient hypotension occurred at the 387 mg/m^2^ dose level, thereby establishing the maximum tolerated dose (MTD) at 298 mg/m^2^. At lower dose levels, the only treatment‐related adverse event observed in more than 10% of patients was fatigue. Following phase II treatment, APTO‐253 was well tolerated and exhibited signs of antitumor activity, with 5 of 21 evaluable patients (23.8%) achieving stable disease, lasting 3.6 to 8.4 months [192]. In addition, in the ongoing Phase Ia/b clinical trial involving patients with relapsed or refractory AML and high‐risk MDS, APTO‐253 was well tolerated across multiple treatment cycles at dose levels of 20, 40, 66, 100, and 150 mg/m^2^. No dose‐limiting toxicities (DLTs) or drug‐related serious adverse events (SAEs) were observed. Only one patient experienced Grade ≥ 3 fatigue, which was probably related to the study drug. However, this trial did not provide efficacy‐related outcome data [193] (Figure 4). Nevertheless, the clinical trial of APTO‐253 was ultimately halted due to safety concerns. The extensive expression and diverse functional roles of KLF4 across multiple somatic cells raise reasonable concerns regarding potential systemic toxicities upon treatment. Furthermore, APTO‐253 acts on multiple molecular targets, including KLF4 and MYC. The potential risk of off‐target effects associated with APTO‐253 may trigger unintended physiological or biochemical responses, representing another major challenge for its clinical translation and application. Exploring strategies to harness KLF4's therapeutic potential while minimizing adverse reactions is needed, which could focus on developing targeted drug delivery systems to enhance tumor‐specific drug accumulation [194]. An additional hurdle lies in the current lack of robust methods for detecting KLF4 in readily accessible body fluids, hindering its immediate application as a liquid biomarker [5]. Addressing these multifaceted challenges is indispensable for the successful clinical translation of KLF4‐targeted therapies.

Generally, transcription factors like KLF4 are often considered “undruggable” due to the lack of well‐defined ligand‐binding sites and protein–protein interaction interfaces, significantly limiting the options for designing KLF4‐targeted drugs [195]. Although KLF4 has been extensively studied in tumors, its potential clinical value in cancer diagnosis and prognosis has yet to be fully established. Future progress hinges on deeper mechanistic investigations and rigorous clinical validation to translate KLF4‐targeted strategies into effective cancer therapies.

## Author Contributions

Conceptualization: M.T. and J.Z.; Literature search: M.T. and J.Z.; Writing – original draft preparation: M.T., J.Z., and B.T.; Writing – review and editing: Q.L., B.T. and M.T.; Visualization: M.T. and B.T.; Supervision: Q.L., B.T. and J.Z., D.M., R.S. All authors have read and approved the final manuscript.

## Funding

This work was supported by the National Natural Science Foundation of China (NO. 82073214, 82473306 to Qi Li).

## Consent

The authors have nothing to report.

## Conflicts of Interest

The authors declare no conflicts of interest.

## Supporting information


**Data S1:** cam471498‐sup‐0001‐supinfo.docx.

## Data Availability

The data that supports the findings of this study are available in the Supporting Information of this article.
