# Supplementary material for: Krüppel‐Like Factor 4, a Hub Gate for Cell Crosstalk in Tumor Microenvironment
Source: Cancer Med. 2026 Jan 14;15(1):e71498. doi: 10.1002/cam4.71498 (PMC12800926; doi:10.1002/cam4.71498)
Supplement: Supplementary file 1 — Data S1: cam471498‐sup‐0001‐supinfo.docx. [file CAM4-15-e71498-s001.docx]

**Supporting Informations**

**The following are the references used in Table 1.**

| Cancer type | role of KLF4 on cancer cells | Molecules regulated by KLF4 | Molecules regulate KLF4 | References |
| --- | --- | --- | --- | --- |
| gastric cancer | suppress | CXCL8 [S1][1]  miR-106a [S2][2]  PODXL [S3][3]  SAMHD1 [S4][4]  TGF-β, Notch, and Wnt signaling pathways [S5][5] | CagA/TET1 [S6][6]  SNHG5/miR-32 [S7][7]  miR-103 [S8][8]  LINC00673 [S9][9]  miR-135b-5p [S10][10] | [S1-S10] |
| lung cancer | suppress | OTUD1 [S11][11]  TIMP3 [S12][12]  SPARC [S13][13]  PLAC8 [S14][14] | miR-25 [S15][15]  LINC00852/hsa-miR-145-5p [S70][16]  TRHDE-AS1/miR-103 [S17][17]  USP10 [S12][12]  miR-3120-5p [S18][18]  PTEN [S19][19]  HDAC [S20][20] | [S11-S20] |
| pancreatic cancer | suppress | LDHA [S21][21]  CD44 [S22][22]  NAG-1, p21 [S23][23]  MSI2 [S24][24]  GPRC5A [S25][25] | MCC-555 [S23][23]  miR-135b-5p [S25][25] | [S21-S25] |
| Colorectal cancer | promote | NDRG2 [S26][26]  p21 [S27][27]  STAT3 [S28][28]  RAB26 [S29][29]  GPA33 [S30][30] | TUG1, EZH2 [S31][31]  TCFL5_E8 [S32][32]  miR-543 [S33][33]  Orlistat [S34][34]  PPARgamma [S10][30] | [S26-S30] |
|  | suppress | MMP2 [S35][35]  miR-153-1 [S31][31]  μ-protocadherin [S36][36]  Bmi1 [S37][37]  IFITM3 [S38][38]  PiHL/EZH2/HMGA2 [S39][39]  RNF112 [S40][40]  RAB26 [S29][29]  HMGB1, hTERT [S41][41] | miR-29a [S35][35]  miR-92a [S42][42]  TUG1, EZH2 [S31][31]  miR-7-5p [S43][43]  miR-103/(and)107 [S44][44]  E2F3/MEX3A [S45][45]  miR-206 [S46][46] | [S35-S46] |
| breast cancer | promote | S100A14 [S47][47]  PFKP [S48][48]  Notch signaling[S49][49] | TPA [S47][47]  MED27 [S27][50]  ATXN3 [S51][51]  FBXO32 [S52][52]  EGR1 [S53][53]  miR-484 [S54][54]  miR-7 [S55][55]  miR-29a [S56][56]  DYRK2/AR [S57][57] | [S47-S57] |
|  | suppress | MMP2 [S58][58]  EGFR [S59][59]  Eralpha [S60][60]  MAPK signaling pathway [S61][61]  E-Cadherin [S62][62] | circEHMT1/miR-1233-3p [S58][58]  DIM [S63][63]  AGPAT9 [S64][64]  Cy3G [S65][65]  DDX3X [S66][66]  TNF-α [S67][67] | [S58-S67] |
| prostate cancer | promote | miR-7 [S68][68]  ARPC5/ADAM17 [S69][69]  PI3K/Akt/p21 [S70][70]  KRT6, KRT13 [S71][71] | miR-148-3p, miR-152-3p [S72][72]  UCA1 [S71][71] | [S68-S72] |
|  | suppress | AKT/p21 signal pathway [S70][70]  SLUG [S73][73] | miR-32-5p [S74][74]  LINC00673 [S75][75] | [S70-S75] |

**Reference**

1 Z. Liu, X. Wu, Y. Tian, W. Zhang, S. Qiao, W. Xu, Y. Liu and S. Wang, H. pylori infection induces CXCL8 expression and promotes gastric cancer progress through downregulating KLF4, Mol Carcinog 60, 524-537 (2021)

2 M. Zhu, N. Zhang and S. He, Transcription factor KLF4 modulates microRNA-106a that targets Smad7 in gastric cancer, Pathol Res Pract 215, 152467 (2019)

3 J. Zhang, Z. Zhu, H. Wu, Z. Yu, Z. Rong, Z. Luo, Y. Xu, K. Huang, Z. Qiu and C. Huang, PODXL, negatively regulated by KLF4, promotes the EMT and metastasis and serves as a novel prognostic indicator of gastric cancer, Gastric Cancer 22, 48-59 (2019)

4 Z. Chen, Z. Jiang, L. Meng, Y. Wang, M. Lin, Z. Wei, W. Han, S. Ying and A. Xu, SAMHD1, positively regulated by KLF4, suppresses the proliferation of gastric cancer cells through MAPK p38 signaling pathway, Cell Cycle 21, 2065-2078 (2022)

5 J. Cui, M. Shi, M. Quan and K. Xie, Regulation of EMT by KLF4 in gastrointestinal cancer, Curr Cancer Drug Targets 13, 986-995 (2013)

6 R. Zhao, Z. Liu, W. Xu, L. Song, H. Ren, Y. Ou, Y. Liu and S. Wang, Helicobacter pylori infection leads to KLF4 inactivation in gastric cancer through a TET1-mediated DNA methylation mechanism, Cancer Med 9, 2551-2563 (2020)

7 L. Zhao, T. Han, Y. Li, J. Sun, S. Zhang, Y. Liu, B. Shan, D. Zheng and J. Shi, The lncRNA SNHG5/miR-32 axis regulates gastric cancer cell proliferation and migration by targeting KLF4, Faseb j 31, 893-903 (2017)

8 J. Zheng, Y. Liu, Y. Qiao, L. Zhang and S. Lu, miR-103 Promotes Proliferation and Metastasis by Targeting KLF4 in Gastric Cancer, Int J Mol Sci 18, (2017)

9 M.C. Ba, H. Long, S.Z. Cui, Y.F. Gong, Z.F. Yan, Y.B. Wu and Y.N. Tu, Long noncoding RNA LINC00673 epigenetically suppresses KLF4 by interacting with EZH2 and DNMT1 in gastric cancer, Oncotarget 8, 95542-95553 (2017)

10 Z. Chen, Y. Gao, S. Gao, D. Song and Y. Feng, MiR-135b-5p promotes viability, proliferation, migration and invasion of gastric cancer cells by targeting Krüppel-like factor 4 (KLF4), Arch Med Sci 16, 167-176 (2020)

11 X. Ma, L. Wang, G. Shi and S. Sun, The deubiquitinase OTUD1 inhibits non-small cell lung cancer progression by deubiquitinating and stabilizing KLF4, Thorac Cancer 13, 761-770 (2022)

12 X. Wang, S. Xia, H. Li, X. Wang, C. Li, Y. Chao, L. Zhang and C. Han, The deubiquitinase USP10 regulates KLF4 stability and suppresses lung tumorigenesis, Cell Death Differ 27, 1747-1764 (2020)

13 Y. Zhou, W.L. Hofstetter, Y. He, W. Hu, A. Pataer, L. Wang, J. Wang, Y. Zhou, L. Yu, B. Fang and S.G. Swisher, KLF4 inhibition of lung cancer cell invasion by suppression of SPARC expression, Cancer Biol Ther 9, 507-513 (2010)

14 Y. Jia, X. Ying, J. Zhou, Y. Chen, X. Luo, S. Xie, Q.C. Wang, W. Hu and L. Wang, The novel KLF4/PLAC8 signaling pathway regulates lung cancer growth, Cell Death Dis 9, 603 (2018)

15 X. Ding, T. Zhong, L. Jiang, J. Huang, Y. Xia and R. Hu, miR-25 enhances cell migration and invasion in non-small-cell lung cancer cells via ERK signaling pathway by inhibiting KLF4, Mol Med Rep 17, 7005-7016 (2018)

16 Z. Tuo, L. Liang and R. Zhou, LINC00852 is associated with poor prognosis in non-small cell lung cancer patients and its inhibition suppresses cancer cell proliferation and chemoresistance via the hsa-miR-145-5p/KLF4 axis, J Gene Med 23, e3384 (2021)

17 B. Zhuan, Y. Lu, Q. Chen, X. Zhao, P. Li, Q. Yuan and Z. Yang, Overexpression of the long noncoding RNA TRHDE-AS1 inhibits the progression of lung cancer via the miRNA-103/KLF4 axis, J Cell Biochem 120, 17616-17624 (2019)

18 H. Xu and Q. Wen, miR‑3120‑5p acts as a diagnostic biomarker in non‑small cell lung cancer and promotes cancer cell proliferation and invasion by targeting KLF4, Mol Med Rep 18, 4621-4628 (2018)

19 G. Lu, Y. Yao, X. Zhang, D. Cui and J. Zhou, Deguelin Attenuates Non-Small-Cell Lung Cancer Cell Metastasis by Upregulating PTEN/KLF4/EMT Signaling Pathway, Dis Markers 2022, 4090346 (2022)

20 T. Yu, X. Chen, W. Zhang, J. Liu, R. Avdiushko, D.L. Napier, A.X. Liu, J.M. Neltner, C. Wang, D. Cohen and C. Liu, KLF4 regulates adult lung tumor-initiating cells and represses K-Ras-mediated lung cancer, Cell Death Differ 23, 207-215 (2016)

21 M. Shi, J. Cui, J. Du, D. Wei, Z. Jia, J. Zhang, Z. Zhu, Y. Gao and K. Xie, A novel KLF4/LDHA signaling pathway regulates aerobic glycolysis in and progression of pancreatic cancer, Clin Cancer Res 20, 4370-4380 (2014)

22 Y. Yan, Z. Li, X. Kong, Z. Jia, X. Zuo, M. Gagea, S. Huang, D. Wei and K. Xie, KLF4-Mediated Suppression of CD44 Signaling Negatively Impacts Pancreatic Cancer Stemness and Metastasis, Cancer Res 76, 2419-2431 (2016)

23 K.W. Min, X. Zhang, T. Imchen and S.J. Baek, A peroxisome proliferator-activated receptor ligand MCC-555 imparts anti-proliferative response in pancreatic cancer cells by PPARgamma-independent up-regulation of KLF4, Toxicol Appl Pharmacol 263, 225-232 (2012)

24 K. Guo, J. Cui, M. Quan, D. Xie, Z. Jia, D. Wei, L. Wang, Y. Gao, Q. Ma and K. Xie, The Novel KLF4/MSI2 Signaling Pathway Regulates Growth and Metastasis of Pancreatic Cancer, Clin Cancer Res 23, 687-696 (2017)

25 D. Liu, Y. Jin, J. Wu, H. Zhu and D. Ye, MiR-135b-5p is an oncogene in pancreatic cancer to regulate GPRC5A expression by targeting transcription factor KLF4, Cell Death Discov 8, 23 (2022)

26 Y. Ma, L. Wu, X. Liu, Y. Xu, W. Shi, Y. Liang, L. Yao, J. Zheng and J. Zhang, KLF4 inhibits colorectal cancer cell proliferation dependent on NDRG2 signaling, Oncol Rep 38, 975-984 (2017)

27 H. Lv, Z. Zhang, Y. Wang, C. Li, W. Gong and X. Wang, MicroRNA-92a Promotes Colorectal Cancer Cell Growth and Migration by Inhibiting KLF4, Oncol Res 23, 283-290 (2016)

28 L. Yuan, Y. Meng and J. Xiang, KLF4 Induces Colorectal Cancer by Promoting EMT via STAT3 Activation, Dig Dis Sci, (2024)

29 Y. Zheng, J. Wu, H. Chen, D. Lin, H. Chen, J. Zheng, H. Xia, L. Huang and C. Zeng, KLF4 targets RAB26 and decreases 5-FU resistance through inhibiting autophagy in colon cancer, Cancer Biol Ther 24, 2226353 (2023)

30 J. Rageul, S. Mottier, A. Jarry, Y. Shah, S. Théoleyre, D. Masson, F.J. Gonzalez, C.L. Laboisse and M.G. Denis, KLF4-dependent, PPARgamma-induced expression of GPA33 in colon cancer cell lines, Int J Cancer 125, 2802-2809 (2009)

31 H. Shao, D. Dong and F. Shao, Long non-coding RNA TUG1-mediated down-regulation of KLF4 contributes to metastasis and the epithelial-to-mesenchymal transition of colorectal cancer by miR-153-1, Cancer Manag Res 11, 8699-8710 (2019)

32 J. Galán-Martínez, K. Stamatakis, I. Sánchez-Gómez, S. Vázquez-Cuesta, N. Gironés and M. Fresno, Isoform-specific effects of transcription factor TCFL5 on the pluripotency-related genes SOX2 and KLF4 in colorectal cancer development, Mol Oncol 16, 1876-1890 (2022)

33 F. Zhai, C. Cao, L. Zhang and J. Zhang, miR-543 promotes colorectal cancer proliferation and metastasis by targeting KLF4, Oncotarget 8, 59246-59256 (2017)

34 M. Noroozi Karimabad, F. Roostaei, M. Mahmoodi and M.R. Hajizadeh, Evaluation of the Effect of Orlistatorlistat on Expression of OCT4, Nanog, SOX2, and KLF4 Genes in Colorectal Cancer SW40 Cell Line, Asian Pac J Cancer Prev 22, 2335-2341 (2021)

35 W. Tang, Y. Zhu, J. Gao, J. Fu, C. Liu, Y. Liu, C. Song, S. Zhu, Y. Leng, G. Wang, W. Chen, P. Du, S. Huang, X. Zhou, J. Kang and L. Cui, MicroRNA-29a promotes colorectal cancer metastasis by regulating matrix metalloproteinase 2 and E-cadherin via KLF4, Br J Cancer 110, 450-458 (2014)

36 S. Parenti, L. Montorsi, S. Fantini, F. Mammoli, C. Gemelli, C.G. Atene, L. Losi, C. Frassineti, B. Calabretta, E. Tagliafico, S. Ferrari, T. Zanocco-Marani and A. Grande, KLF4 Mediates the Effect of 5-ASA on the β-Catenin Pathway in Colon Cancer Cells, Cancer Prev Res (Phila) 11, 503-510 (2018)

37 T. Yu, X. Chen, W. Zhang, D. Colon, J. Shi, D. Napier, P. Rychahou, W. Lu, E.Y. Lee, H.L. Weiss, B.M. Evers and C. Liu, Regulation of the potential marker for intestinal cells, Bmi1, by β-catenin and the zinc finger protein KLF4: implications for colon cancer, J Biol Chem 287, 3760-3768 (2012)

38 D. Li, Z. Peng, H. Tang, P. Wei, X. Kong, D. Yan, F. Huang, Q. Li, X. Le, Q. Li and K. Xie, KLF4-mediated negative regulation of IFITM3 expression plays a critical role in colon cancer pathogenesis, Clin Cancer Res 17, 3558-3568 (2011)

39 X. Deng, F. Kong, S. Li, H. Jiang, L. Dong, X. Xu, X. Zhang, H. Yuan, Y. Xu, Y. Chu, H. Peng and M. Guan, A KLF4/PiHL/EZH2/HMGA2 regulatory axis and its function in promoting oxaliplatin-resistance of colorectal cancer, Cell Death Dis 12, 485 (2021)

40 C. Li, W. Guan, D. Geng and Y. Feng, RNF112, whose transcription is regulated by KLF4, inhibits colorectal cancer growth via promoting ubiquitin-dependent degradation of NAA40, Cell Biol Toxicol 41, 22 (2025)

41 S.S. Yadav, M. Kumar, A. Varshney and P.K. Yadava, KLF4 sensitizes the colon cancer cell HCT-15 to cisplatin by altering the expression of HMGB1 and hTERT, Life Sci 220, 169-176 (2019)

42 M. Zhou, S. Wang, D. Liu and J. Zhou, LINC01915 Facilitates the Conversion of Normal Fibroblasts into Cancer-Associated Fibroblasts Induced by Colorectal Cancer-Derived Extracellular Vesicles through the miR-92a-3p/KLF4/CH25H Axis, ACS Biomater Sci Eng 7, 5255-5268 (2021)

43 Y. Shang, Z. Zhu, Y. Zhang, F. Ji, L. Zhu, M. Liu, Y. Deng, G. Lv, D. Li, Z. Zhou, B. Lu and C.G. Fu, MiR-7-5p/KLF4 signaling inhibits stemness and radioresistance in colorectal cancer, Cell Death Discov 9, 42 (2023)

44 H.Y. Chen, Y.M. Lin, H.C. Chung, Y.D. Lang, C.J. Lin, J. Huang, W.C. Wang, F.M. Lin, Z. Chen, H.D. Huang, J.Y. Shyy, J.T. Liang and R.H. Chen, miR-103/107 promote metastasis of colorectal cancer by targeting the metastasis suppressors DAPK and KLF4, Cancer Res 72, 3631-3641 (2012)

45 X. Yang, G. Li, Y. Tian, X. Wang, J. Xu, R. Liu, M. Deng, C. Shao, Y. Pan, X. Wu, M. Li, C. Zhang, R. Liu, J. Qin, C. Zhang, Z. Liu, X. Wu, M.V. Plikus, C.J. Lengner, Z. Zheng, C. Lv and Z. Yu, Identifying the E2F3-MEX3A-KLF4 signaling axis that sustains cancer cells in undifferentiated and proliferative state, Theranostics 12, 6865-6882 (2022)

46 M.A. Parasramka, W.M. Dashwood, R. Wang, H.H. Saeed, D.E. Williams, E. Ho and R.H. Dashwood, A role for low-abundance miRNAs in colon cancer: the miR-206/Krüppel-like factor 4 (KLF4) axis, Clin Epigenetics 4, 16 (2012)

47 H. He, S. Li, H. Chen, L. Li, C. Xu, F. Ding, Y. Zhan, J. Ma, S. Zhang, Y. Shi, C. Qu and Z. Liu, 12-O-tetradecanoylphorbol-13-acetate promotes breast cancer cell motility by increasing S100A14 level in a Kruppel-like transcription factor 4 (KLF4)-dependent manner, J Biol Chem 289, 9089-9099 (2014)

48 J.S. Moon, H.E. Kim, E. Koh, S.H. Park, W.J. Jin, B.W. Park, S.W. Park and K.S. Kim, Krüppel-like factor 4 (KLF4) activates the transcription of the gene for the platelet isoform of phosphofructokinase (PFKP) in breast cancer, J Biol Chem 286, 23808-23816 (2011)

49 F. Yu, J. Li, H. Chen, J. Fu, S. Ray, S. Huang, H. Zheng and W. Ai, Kruppel-like factor 4 (KLF4) is required for maintenance of breast cancer stem cells and for cell migration and invasion, Oncogene 30, 2161-2172 (2011)

50 R. Wang, W. Yu, T. Zhu, F. Lin, C. Hua, L. Ru, P. Guo, X. Wan, G. Xue, Z. Guo, S. Han, K. Lv, G. Zhang, H. Ge, W. Guo, L. Xu and W. Deng, MED27 plays a tumor-promoting role in breast cancer progression by targeting KLF4, Cancer Sci 114, 2277-2292 (2023)

51 H. Zou, H. Chen, Z. Zhou, Y. Wan and Z. Liu, ATXN3 promotes breast cancer metastasis by deubiquitinating KLF4, Cancer Lett 467, 19-28 (2019)

52 H. Zhou, Y. Liu, R. Zhu, F. Ding, Y. Wan, Y. Li and Z. Liu, FBXO32 suppresses breast cancer tumorigenesis through targeting KLF4 to proteasomal degradation, Oncogene 36, 3312-3321 (2017)

53 E. Jung, Y.H. Lee, S. Ou, T.Y. Kim and S.Y. Shin, EGR1 Regulation of Vasculogenic Mimicry in the MDA-MB-231 Triple-Negative Breast Cancer Cell Line through the Upregulation of KLF4 Expression, Int J Mol Sci 24, (2023)

54 Y. Wei, H. Li and Q. Qu, miR-484 suppresses endocrine therapy-resistant cells by inhibiting KLF4-induced cancer stem cells in estrogen receptor-positive cancers, Breast Cancer 28, 175-186 (2021)

55 H. Okuda, F. Xing, P.R. Pandey, S. Sharma, M. Watabe, S.K. Pai, Y.Y. Mo, M. Iiizumi-Gairani, S. Hirota, Y. Liu, K. Wu, R. Pochampally and K. Watabe, miR-7 suppresses brain metastasis of breast cancer stem-like cells by modulating KLF4, Cancer Res 73, 1434-1444 (2013)

56 Q. Zhao, Y. Shang, J. Lü, Y. Liu, T. Wang, D. Li, J. Li, Y. Lu, Z. Wang and Z. Yu, miR-29a-KLF4 signaling inhibits breast tumor initiation by regulating cancer stem cells, Int Immunopharmacol 130, 111797 (2024)

57 R. Mimoto, Y. Imawari, S. Hirooka, H. Takeyama and K. Yoshida, Impairment of DYRK2 augments stem-like traits by promoting KLF4 expression in breast cancer, Oncogene 36, 1862-1872 (2017)

58 M. Lu, Y. Wu, B. Zeng, J. Sun, Y. Li, J. Luo, L. Wang, Z. Yi, H. Li and G. Ren, CircEHMT1 inhibits metastatic potential of breast cancer cells by modulating miR-1233-3p/KLF4/MMP2 axis, Biochem Biophys Res Commun 526, 306-313 (2020)

59 M.S. Roberts, L.J. Anstine, V.S. Finke, B.L. Bryson, B.M. Webb, K.L. Weber-Bonk, D.D. Seachrist, P.R. Majmudar and R.A. Keri, KLF4 defines the efficacy of the epidermal growth factor receptor inhibitor, erlotinib, in triple-negative breast cancer cells by repressing the EGFR gene, Breast Cancer Res 22, 66 (2020)

60 K. Akaogi, Y. Nakajima, I. Ito, S. Kawasaki, S.H. Oie, A. Murayama, K. Kimura and J. Yanagisawa, KLF4 suppresses estrogen-dependent breast cancer growth by inhibiting the transcriptional activity of ERalpha, Oncogene 28, 2894-2902 (2009)

61 Y. Jia, J. Zhou, X. Luo, M. Chen, Y. Chen, J. Wang, H. Xiong, X. Ying, W. Hu, W. Zhao, W. Deng and L. Wang, KLF4 overcomes tamoxifen resistance by suppressing MAPK signaling pathway and predicts good prognosis in breast cancer, Cell Signal 42, 165-175 (2018)

62 H.K. Lee, D.S. Lee and J.C. Park, Nuclear factor I-C regulates E-cadherin via control of KLF4 in breast cancer, BMC Cancer 15, 113 (2015)

63 F. Xiang, Z. Zhu, M. Zhang, J. Wang, Z. Chen, X. Li, T. Zhang, Q. Gu, R. Wu and X. Kang, 3,3'-Diindolylmethane Enhances Paclitaxel Sensitivity by Suppressing DNMT1-Mediated KLF4 Methylation in Breast Cancer, Front Oncol 11, 627856 (2021)

64 S.H. Fan, Y.Y. Wang, Z.Y. Wu, Z.F. Zhang, J. Lu, M.Q. Li, Q. Shan, D.M. Wu, C.H. Sun, B. Hu and Y.L. Zheng, AGPAT9 suppresses cell growth, invasion and metastasis by counteracting acidic tumor microenvironment through KLF4/LASS2/V-ATPase signaling pathway in breast cancer, Oncotarget 6, 18406-18417 (2015)

65 D. Chen, M. Yuan, Q. Ye, X. Wang, J. Xu, G. Shi and Z. Hu, Cyanidin-3-O-glucoside inhibits epithelial-to-mesenchymal transition, and migration and invasion of breast cancer cells by upregulating KLF4, Food Nutr Res 64, (2020)

66 E. Cannizzaro, A.J. Bannister, N. Han, A. Alendar and T. Kouzarides, DDX3X RNA helicase affects breast cancer cell cycle progression by regulating expression of KLF4, FEBS Lett 592, 2308-2322 (2018)

67 C. Liu, C. Feng, H. Li, E. Zhang, B. Liu, Y. Wang and P. Wang, The mechanisms of tumor necrosis factor α in regulating Krüpple-like factor 4 expression in SK-BR-3 breast cancer cells, Asia Pac J Clin Oncol 21, 123-128 (2025)

68 L.Z. Wei, Y.Q. Wang, Y.L. Chang, N. An, X. Wang, P.J. Zhou, H.H. Zhu, Y.X. Fang and W.Q. Gao, Imbalance of a KLF4-miR-7 auto-regulatory feedback loop promotes prostate cancer cell growth by impairing microRNA processing, Am J Cancer Res 8, 226-244 (2018)

69 G. Qu, Y. Zhang, H. Duan, C. Tang, G. Yang, D. Chen and Y. Xu, ARPC5 is transcriptionally activated by KLF4, and promotes cell migration and invasion in prostate cancer via up-regulating ADAM17 : ARPC5 serves as an oncogene in prostate cancer, Apoptosis 28, 783-795 (2023)

70 Y.L. Chang, P.J. Zhou, L. Wei, W. Li, Z. Ji, Y.X. Fang and W.Q. Gao, MicroRNA-7 inhibits the stemness of prostate cancer stem-like cells and tumorigenesis by repressing KLF4/PI3K/Akt/p21 pathway, Oncotarget 6, 24017-24031 (2015)

71 X.Y. Na, Z.Y. Liu, P.P. Ren, R. Yu and X.S. Shang, Long non-coding RNA UCA1 contributes to the progression of prostate cancer and regulates proliferation through KLF4-KRT6/13 signaling pathway, Int J Clin Exp Med 8, 12609-12616 (2015)

72 F. Feng, H. Liu, A. Chen, Q. Xia, Y. Zhao, X. Jin and J. Huang, miR-148-3p and miR-152-3p synergistically regulate prostate cancer progression via repressing KLF4, J Cell Biochem 120, 17228-17239 (2019)

73 Y.N. Liu, W. Abou-Kheir, J.J. Yin, L. Fang, P. Hynes, O. Casey, D. Hu, Y. Wan, V. Seng, H. Sheppard-Tillman, P. Martin and K. Kelly, Critical and reciprocal regulation of KLF4 and SLUG in transforming growth factor β-initiated prostate cancer epithelial-mesenchymal transition, Mol Cell Biol 32, 941-953 (2012)

74 L. Zhang, X. Li, Y. Chao, R. He, J. Liu, Y. Yuan, W. Zhao, C. Han and X. Song, KLF4, a miR-32-5p targeted gene, promotes cisplatin-induced apoptosis by upregulating BIK expression in prostate cancer, Cell Commun Signal 16, 53 (2018)

75 Z. Jiang, Y. Zhang, X. Chen, P. Wu and D. Chen, Long non-coding RNA LINC00673 silencing inhibits proliferation and drug resistance of prostate cancer cells via decreasing KLF4 promoter methylation, J Cell Mol Med 24, 1878-1892 (2020)
